# Mechanoregulated trabecular bone adaptation: Progress report on *in silico* approaches

**DOI:** 10.1016/j.bbiosy.2022.100058

**Published:** 2022-07-21

**Authors:** Ekaterina Smotrova, Simin Li, Vadim V. Silberschmidt

**Affiliations:** Wolfson School of Mechanical, Electrical and Manufacturing Engineering, Loughborough University, Loughborough, Leics LE11 3TU, UK

**Keywords:** Trabecular bone, Bone adaptation, *In silico* modelling, BA, Bone adaptation, BMD, Bone mineral density, BR, Bone remodelling, BV/TV, Bone volume fraction, FE, Finite element, LCN, Lacunar-canalicular network, SED, Strain energy density, TB, Trabecular bone

## Abstract

*Adaptation* is the process by which bone responds to changes in loading environment and modulates its properties and spatial organization to meet the mechanical demands. Adaptation in trabecular bone is achieved through increase in bone mass and alignment of trabecular-bone morphology along the loading direction. This transformation of internal microstructure is governed by mechanical stimuli sensed by mechanosensory cells in the bone matrix. Realisation of adaptation in the form of local bone-resorption and -formation activities as a function of mechanical stimuli is still debated. *In silico* modelling is a useful tool for simulation of various scenarios that cannot be investigated *in vivo* and particularly well suited for prediction of trabecular bone adaptation. This progress report presents the recent advances in *in silico* modelling of mechanoregulated adaptation at the scale of trabecular bone tissue. Four well-established bone-adaptation models are reviewed in terms of their recent improvements and validation. They consider various mechanical factors: (i) strain energy density, (ii) strain and damage, (iii) stress nonuniformity and (iv) daily stress. Contradictions of these models are discussed and their ability to describe adequately a real-life mechanoregulation process in bone is compared.

## Highlights

• Recent advances *in silico* modelling of mechanoregulated adaptation at tissue level.
• Four groups of bone adaptation models are discussed and compared.
• Their ability to predict mechanoregulation processes in bone is analysed.

## 1 Introduction

Bone is a living material, which demonstrates continuously changing structural configurations and mechanical properties. Although many engineering materials are mechanically superior, bone is unique in its ability to respond to changes in its external loading environment through the process of *bone adaptation* (BA). Adaptation in trabecular bone (TB) – the spongy component of many long bones – is characterised by changes in mechanical properties of this bone tissue and alignment of trabecular load-bearing architecture along the loading direction. The ability to predict TB adaptation to mechanical loads in terms of local changes in bone mineral density (BMD) and microarchitecture can provide an opportunity to simulate the effects of load-induced structural alterations caused by various conditions, such as altering strain magnitudes/directions in femur after total hip replacement, the effect of exercise in osteoporosis prevention and disuse-induced bone mass loss in micro-gravity conditions of spaceflights [1], [2], [3].

A combined use of mathematical models and computer simulations, the so-called *in silico* approach, offers many important benefits for investigation of patient-specific changes in structure and mechanical properties of TB in otherwise inaccessible clinical conditions [4]. Several *in silico* approaches were proposed recently to predict the load-induced BA at different scales of bone hierarchy. At the cellular level, models primarily aim at describing activities of bone-forming and -resorbing cells and the underlying pathways of biochemical and mechanical signalling (e.g., [5]). In contrast, at the bone-tissue level, multiple models exist that predict changes in trabecular BMD and microarchitecture based on such principles as direct mechanoregulation [6], [7], [8], [9], topology optimization [10,11] and homogenisation [12,13]. Other approaches, such as those based on thermodynamics of irreversible process, were also developed to simulate the anisotropic remodelling and growth of TB [14,15]. Within the domain of continuum mechanics, mechanoregulation-based models, being the subject of this paper, mathematically describe the modulation of TB adaptation due to mechanical stimuli. They consider TB as either a continuous material with specific values of density and Young's modulus, neglecting the trabecular microarchitecture, or as a complex inhomogeneous material, incorporating individual elements of this bone tissue, the *trabeculae*. This report details the recent advances in prediction of BA using *in silico* mechanoregulation-based approaches. In addition, contradictions of selected well-established models of BA are discussed and their ability to predict the load-induced turnover in TB is compared.

### 1.1 Trabecular bone remodelling and adaptation

Made up mostly of cells, collagenous mineralized extracellular matrix and water, bone is a living organ that adapts to mechanical stresses through resorption of old or damaged tissue and formation of new bone material via the activities of osteoclast and osteoblast cells, respectively, – the process known as *bone remodelling* (BR). As more bone matrix is formed by the osteoblasts, they become encapsulated in their secretions and develop into osteocytes. Osteocytes are contained within the pores in the bone matrix, called *lacunae*, and connect with each other and with the bone surface via tiny canaliculi, forming a 3D lacunar-canalicular network (LCN). Osteocytes are hypothesised to sense local mechanical signals and, based on these, coordinate the action of osteoclasts and osteoblasts on the nearest bone surface. Driven by the load-induced stimuli, respective bone cells resorb and deposit bone tissue in a coordinated manner aiming for adaptation of structural-functional properties of trabeculae to meet the mechanical demands of bone [16,17].

Loading, that the skeleton is subjected to in daily activities, modulates mechanical properties and spatial organisation of TB. Intensified mechanical use results in thicker and mechanically stronger bones. For example, impact-based physical activities such as volleyball and combat sports resulted in higher BMD in young adults as compared to no exercise [18]. High-impact dynamic exercise improved structural and compositional parameters of TB in military recruits [19] and postmenopausal women [20]. In contrast, a lack of mechanostimulation induces a decrease in the bone mass and thinning of trabeculae. For instance, significant deterioration of TB's morphological properties was observed in 3-4 weeks of bone immobilization in animals [21,22]. However, despite this abundant evidence of load-induced BA, the exact nature of the mechanical stimulus triggering the remodelling activities is still unknown. Several mechanical quantities were proposed as candidates for the BA stimuli; they are discussed below.

### 1.2 Mechanical stimuli of bone adaptation

*Strain energy density* (SED) was among the early factors considered to drive BA. The pioneering work of Fyhrie et al. [23] mathematically described a theory of TB optimising its density and orientation to minimise the strain energy, causing formation of the least material for a given load. This theory was supported by combined experimental and computational studies, which demonstrated that the SED magnitude correlated well with the local areas of bone resorption and formation [24], [25], [26], [27], [28]. However, in another study [29], SED proved less effective than interstitial fluid flow at predicting the BA in double-labelled histological sections of loaded murine tibiae.

*Microcracks due to excessive bone straining* are hypothesised to be another trigger of BR. There is experimental evidence of fatigue microdamage leading to ruptures of the osteocytes processes and apoptosis of osteocytes near damaged regions [30]. Such damage to a living material initiates production of osteoclastogenetic signals by the neighboring osteocytes, thus triggering bone resorption [31]. In addition, some microdamage was present in bone in normal conditions of mechanical use and accumulated when the rate of microcrack-formation exceeded the rate of damage removal (e.g., in severe loading) [32]. Finite-element (FE) studies [33,34] demonstrated beneficial results in analysis of changes in local stress fields in bone matrix caused by microdamage that can be sensed by osteocytes, thus providing a theoretical support to the experimental observations of damage-induced BR. The amount of microdamage can therefore serve as an important indicator of the resorption activities in bone and is a suitable candidate for the BA stimulus.

Based on a theory of a local equi-stress state as the optimal condition at the remodelling equilibrium [35], Adachi et al. [36] proposed a *stress nonuniformity* mechanical stimulus as a driving force of BA. To support this hypothesis, the authors compared local levels of stimuli (SED and von Mises stress) and those distributed over the neighbouring bone cells (SED integration and stress nonuniformity) by measuring the distribution patterns of these stimuli on the trabecular compartment of rat vertebrae under three simulated loading cases [37]. For all cases, the nonuniformity of von Mises stress stimulus demonstrated the most smooth and symmetric distribution function, when plotted as frequency of stimulus against its normalized magnitude. According to the authors, these features make the nonuniformity of von Mises stress the more likely candidate for the BA stimuli.

Another approach to determine the BA stimulus was based on bone's loading history rather than on instantaneous values of mechanical stimuli. By combining a cyclic stress magnitude and a number of stress cycles during the day, Carter et al. [38] proposed a *daily stress* stimulus to regulate BA. The importance of considering the cyclic loading in regulation of BA was supported by the observation that only dynamic strain [39,40], not static one [41], increased the bone mass and strength.

Although there is no consensus in the literature regarding the mechanical stimulus that initiates BA, several mathematical models were proposed for prediction of mechanoregulated BR. Analysis of the literature demonstrated that there are four main groups of BA models that directly regulate TB density and microarchitecture by using one of the following mechanical stimuli: (i) SED; (ii) strain (and related damage), (iii) stress nonuniformity; and (iv) daily stress (see details in the discussion above). From each group one original well-established model was selected for in-depth analysis. The rest of the algorithms in each group have only some minor variations and modifications compared to the selected models. The following section presents numerical implementations of the four selected BA models together with the review of their recent improvements and validation.

## 2 Numerical implementations of bone adaptation models

**Fig. 1:**
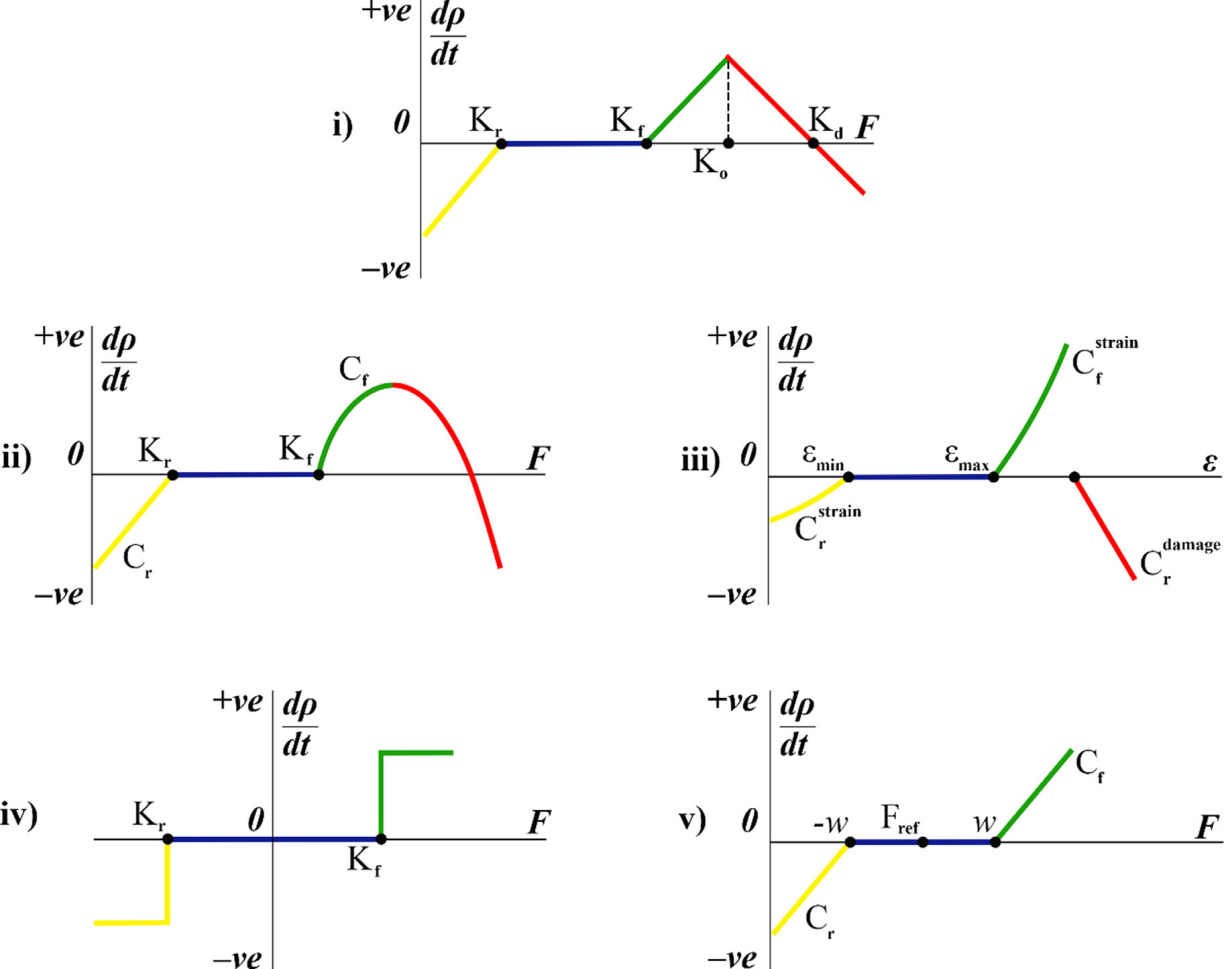
BR rate *d*ρ/*dt* against stimuli in mechanostat theory and four groups of BA models: (i) mechanostat theory (*F* – BR stimulus); (ii) Model A: strain energy density model (*F* – SED); (iii) Model B: strain and damage model (ε – strain); (iv) Model C: stress nonuniformity model (*F* – stress nonuniformity); (v) Model D: daily stress model (*F* – daily stress) (see Table 1 for description of the parameters). Regions of the BR curves: yellow – resorption due to low stimulus; blue – lazy zone; green – formation due to high stimulus; red – overload-induced resorption.

This report reviews four BA models named according to the stimuli used: strain energy density – *Model A*; strain and damage – *Model B*; stress nonuniformity – *Model C*; daily stress – *Model D*. These models are described with differential equations relating specific mechanical stimuli to the changes in BMD and the trabeculae shape. However, the underlying concept of the reviewed models can be generalized using the well-known “mechanostat” theory of Frost [42] that describes the rate of BR with respect to a mechanical stimulus *F* (Fig. 1 (i)). An initial region of the remodelling curve denotes bone resorption at low values of the mechanical stimulus up to *K_r_*. As the load increases, the BR rate reaches a quiescent state or a “lazy zone”, where bone resorption is equally compensated by bone formation and no change in local bone density occurs (*K_r_* < *F* < *K_f_*). When mechanical stimulus exceeds the bone formation threshold *K_f_*, bone deposition prevails over resorption, producing a rising slope in the remodelling curve. Recent experimental evidence of increased resorption near regions of microdamage [43] resulted in the updated curve by adding the parts representing extreme over-straining and microdamage accumulation (*F* > *K_o_* and *F* > *K_d_*, respectively), when the rate of bone formation cannot compensate intensified resorption.

Obviously, the authors of the following models used different notations for parameters of their models. Here we present them in the unified form (Fig. 1 and Table 1).

**Table 1:** Numerical implementation of BA models.

Model	Mechanical stimulus *S*	Mechanical stimulus *F* at site of remodelling	Mechanical stimulus decay function *f*	Rate of density change *dρ/dt*
A	$S_{j}=\frac{U_{j}}{\rho_{j}}=\frac{{\overset{↔}{\varepsilon }}_{j}{\overset{↔}{\sigma }}_{j}}{2\rho_{j}}$ Eq. (1)	$F_{x}=\sum_{j=1}^{i}\mu_{j}S_{j}f_{j}\left(d\right)$ Eq. (2)	$f\left(d\right)=exp\left(\frac{-d}{d_{0}}\right)$ Eq. (3)	$\frac{d\rho }{dt}=\left\{ \begin{array}{cc} C_{r}\left(F_{x}-K_{r}\right) & (F_{x}<K_{r}),\\ 0 & (K_{r}<F_{x}<K_{f}),\\ C_{f}\left(F_{x}-K_{f}\right)-r_{ovl} & (F_{x}>K_{f}). \end{array}\right.$ Eq. (4)
				$\text{where}\;r_{ovl}=D{\left(F_{x}-K_{f}\right)}^{2}$ Eq. (5)
B	See Eq. (1)	$F_{x}=\sum_{j=1}^{i}\left(S_{j}-S_{ref}\right)f_{j}\left(d\right)$ Eq. (6)	See Eq. (3)	$\frac{d\rho }{dt}=\left\{ \begin{array}{cc} C_{r}^{strain}F_{x} & (\varepsilon_{x}<\varepsilon_{min}),\\ 0 & (\varepsilon_{min}<\varepsilon_{x}<\varepsilon_{max}),\\ C_{f}^{strain}F_{x} & (\omega_{x}<\omega_{crit}\;\;and\;\;\varepsilon_{x}>\varepsilon_{max}),\\ C_{r}^{dmg}\left(\omega_{x}-\omega_{crit}\right) & \left(\omega_{x}>\omega_{crit}\right) \end{array}\right.$ Eq. (8)
		$\begin{array}{c} \text{where}\\ S_{ref}=\left\{ \begin{array}{cc} \frac{{\overset{↔}{\varepsilon }}_{min}{\overset{↔}{\sigma }}_{j}}{2\rho_{j}} & (\varepsilon_{j}<\varepsilon_{min}),\\ S_{j} & (\varepsilon_{min}<\varepsilon_{x}<\varepsilon_{max}),\\ \frac{{\overset{↔}{\varepsilon }}_{max}{\overset{↔}{\sigma }}_{j}}{2\rho_{j}} & (\varepsilon_{j}>\varepsilon_{max}). \end{array}\right. \end{array}$ Eq. (7)		$\text{where}\;\;\omega_{x}=\frac{\sum n}{N_{f}}$ Eq. (9)
C	$S_{j}=\sigma_{j}$ Eq. (10)	$F_{x}=\ln\frac{S_{x}{\int \;}_{A}f_{j}\left(d\right)dA}{{\int \;}_{A}f_{j}\left(d\right)S_{j}dA\;}$ Eq. (11)	$f\left(d\right)=1-\frac{d}{l}$ Eq. (12)	$\frac{d\rho }{dt}=\left\{ \begin{array}{cc} -1 & (F_{x}<K_{r}),\\ 0 & (K_{r}<F_{x}<K_{f}),\\ 1 & (F_{x}>K_{f}). \end{array}\right.$ Eq. (13)
D	$S_{j}=\sqrt{2E_{j}U_{j}}$ Eq. (14)	$F_{x}={\left(\sum_{day}n{\left(S_{x}\right)}^{m}\right)}^{1/m}$ Eq. (15)	N/A	$\frac{d\rho }{dt}=\left\{ \begin{array}{cc} C_{r}\left((F_{x}-F_{ref})+w\right) & ((F_{x}-F_{ref})<-w),\\ 0 & (-w⩽(F_{x}-F_{ref})⩽w),\\ C_{f}\left((F_{x}-F_{ref})+w\right) & ((F_{x}-F_{ref})>w). \end{array}\right.$ Eq. (16)

*Parameters common for models*:

*C_f_* – formation slope, *C_r_* – resorption slope, *d* – distance between sensor cell *j* and site of remodelling *x, d*_0_ – distance from sensor cell *j*, at which stimulus magnitude is reduced to 37%, *E* – Young's modulus, *f* – stimulus decay function, *i* – number of sensor cells, *j* – sensor cell, *K_f_* – bone-formation threshold, *K_r_* – bone-resorption threshold, *n* – number of cycles at given stress, *U* – SED, *x* – site of remodelling, $\overset{↔}{\varepsilon }$ – strain tensor, ρ – density of material, $\overset{↔}{\sigma }$ – stress tensor.

*Parameters specific for models*:

Model A (after [6,[44], [45], [46]]): *F* – SED stimulus at site of remodelling, *D* – constant of overload resorption, *r_ovl_* – amount of bone removed by osteoclasts due to overload, *S* – SED stimulus, μ – mechanosensitivity of osteocyte.

Model B (after [7,47,48]): $C_{f}^{strain}$ – formation slope for strain-related stimulus, $C_{r}^{dmg}$ – resorption slope for damage-related stimulus, $C_{r}^{strain}$ – resorption slope for strain-related stimulus, *F* – strain-related stimulus at site of remodelling, *N_f_* – number of cycles to failure for material at given stress, *S* – SED stimulus, *S_ref_* – reference mechanical stimulus, ε – strain, ε_*max*_ and ε_*min*_ – values of strain at borders of lazy zone, ω – accumulated damage, ω_*crit*_ – critical value of damage.

Model C (after [8,36,49]): *A* – trabecular surface, *F* – stress nonuniformity stimulus at site of remodelling, *l* – osteocyte influence distance, *S* – stress stimulus, σ – stress.

Model D (after [9,50,51]): *F* – daily stress stimulus at site of remodelling, *F_ref_* – reference daily stress stimulus, *m* – experimentally defined constant, *S* – effective stress stimulus, *w* – half-width of lazy zone.

### 2.1 Model A – Strain energy density

The model of Huiskes et al. [44] differentiates bone cells into two types: (i) *sensor cells* (osteocytes) located throughout the bone material that sense mechanical stimuli and transmit them to the (ii) *actor cells* (osteoblasts and osteoclasts) concentrated on the bone surface. Mechanical stimulus triggering BA is based on SED calculated as given by Eq. (1) (Table 1). The value of stimulus at the actor cell is the weighted sum of the SED-based stimuli transmitted from the neighbouring sensor cells (Eq. (2)). The authors introduced the dependence of the stimulus transmitted from the sensor cell to the actor cell, on the distance between these cells. This dependence was expressed by an exponential decay function (Eq. (3)), with the stimulus magnitude reduced as it was transmitted from the osteocyte to the actor cell within the osteocyte influence distance. The value of osteocyte influence distance varied in the studies of Huiskes and colleagues [6,52,53] in the range of 200–400 µm. If the total stimulus at the actor cell exceeds the bone formation threshold, the local bone deposition starts. Bone resorption is triggered when the total stimulus is below the bone-resorption threshold. For the deposition and resorption processes, the local change in bone density is given by Eq. (4).

Model A was used in many studies to predict BA. For example, when applied to 2D FE models of osteopetrotic (a rare bone disorder resulting in abnormal bone growth and increase in TB volume) and reduced mineralization TB subjected to loading, this algorithm demonstrated 63% and 23% increases in bone volume fraction (BV/TV), respectively, that is in general agreement with the literature data (18–80)% [54]. The described model was also used to predict the BMD distribution in reconstructed human mandibles [55]. Regression analysis revealed good correlation (0.7) between the simulated BMD and longitudinal *in vivo* CT scans. In addition, Model A was applied to loaded healthy and osteoporotic FE models of mice vertebrae, demonstrating 98% and 94% correspondence in BV/TV and trabecular thickness, respectively, against *in vivo* micro-CT scans [56,57].

Model A was extended by introduction of an overload-induced resorption term (*r_ovl_*) into Eq. (4), producing the remodelling curve shown in Fig. 1(ii) [45,46]. In addition, the described model incorporated a reduction of the osteocyte influence distance in the overload stage [58], based on experimental evidence of microdamage disconnecting the LCN [30], thus inhibiting transduction of mechanical stimuli. However, interpretation of the results was limited by the non-biological origin of the trabecular lattice used in this study.

### 2.2 Model B – Strain and damage

The model proposed by McNamara et al. [7] (Fig. 1(iii)) represents BA using two stimuli: strain and accumulated damage. Similar to the algorithm presented above, this model calculates the strain-related mechanical stimulus based on SED (Eq. (1)). At the actor cell the stimuli transmitted from neighbouring osteocytes are summated to give the total value of stimulus at this cell (Eq. (6)). This model implements the same attenuation function as does Model A, with the magnitude of stimulus transmitted between two cells exponentially decreasing with the distance between them (Eq. (3)). Accumulated damage is represented by a ratio of the number of cycles undergone by the bone at a particular stress to the number of cycles to failure at that stress (Eq. (9)). Driven by both strain- and damage-related stimuli, the local bone density changes according to Eq. (8).

The described model was continuously updated by Prendergast and colleagues [47,48], with many efforts in the last decade focused on validation of this algorithm against experimental data. For instance, when applied to an osteoporotic vertebral body model subjected to loading, it demonstrated a 29% decrease in BV/TV compared to the healthy model [59], which is close to the experimental result for human vertebrae (25%) [60]. In addition, Model B was used for prediction of BMD distributions in a FE femur model after implantation of a hip prosthesis [61,62]. The adaptation patterns obtained corresponded to clinical observations. The limitation of the last two studies was simulation of a simplified trabecular geometry only, with the whole bone modelled as isotropic and spatially homogeneous material. In addition, only one loading case that corresponded to the maximum hip-joint loading during walking was analysed.

### 2.3 Model C – Stress nonuniformity

**Fig. 2:**
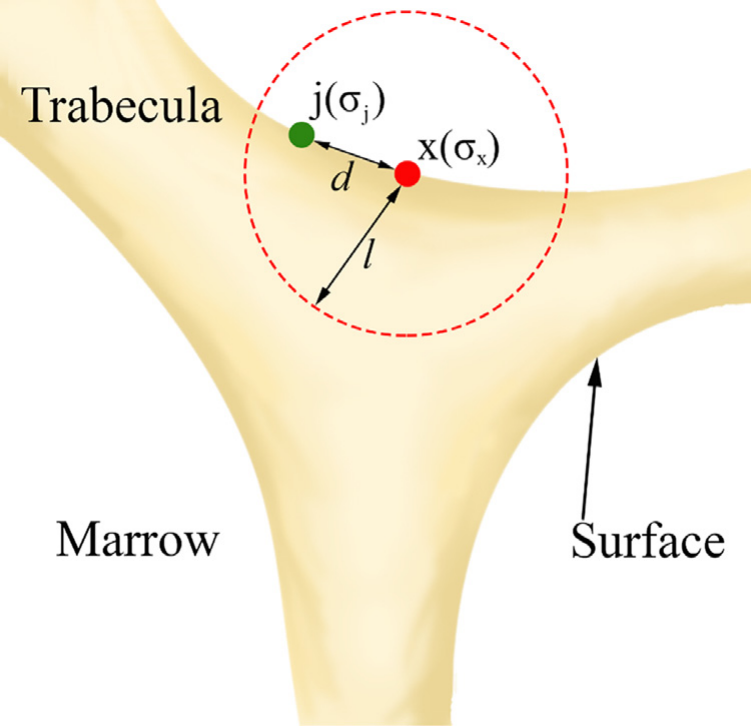
Parameters of Model C [8] (see Table 1 for description of parameters).

The adaptation model of Adachi et al. [8,36] uses nonuniformity of local stress environment as a stimulus to regulate the adaptation process in bone (Eq. (11)). In contrast to Models A and B, this model uses a linear decay function of the stimulus, transmitted to the bone surface cell, within the osteocyte influence distance (Eq. (12) and Fig. 2). The value of osteocyte influence distance varied in the studies of Adachi in the range of 250–1000 μm [8,49,63].

This model assumed that remodelling only happened at the bone surface. The local bone remodelling rate was defined using a step function (Fig. 1(iv)) with the following characteristic regions: (i) bone resorption when the stress nonuniformity stimulus is below the resorption threshold; (ii) lazy zone corresponding to the absence of BR when the stimulus lies between the resorption and formation thresholds; (iii) bone formation when the stress nonuniformity stimulus exceeds the formation threshold. This function is mathematically expressed by Eq. (13).

Model C was implemented for prediction of TB adaptation in proximal femur caused by such loading conditions as one-legged stance, abduction and adduction [63]. The simulation results demonstrated the alignment of trabeculae, expressed as fabric ellipsoids [64], along the direction of principal stresses. Furthermore, the described model was used to predict the adaptation in healthy and osteoporotic porcine TB [65,66]. The pathological bone condition was modelled by changing the values of formation and resorption thresholds that corresponded to different sensitivities of osteocytes in detection of the mechanical stimulus. Although not validated against longitudinal CT scans yet, these studies predicted a 30% loss in the Young's modulus and BV/TV in the adapted osteoporotic model as compared to the healthy model, which is comparable with the values reported in the literature: 25–70% [67] and 10–20% [68], respectively.

This adaptation model was further modified to analyse the effect of cyclic loading on BA [69,70]. Loading-induced fluid flow through the LCN as observed *ex vivo* in human biopsies [71] and *in vivo* in mice [72] was incorporated into Model C. The shear stress on osteocytes induced by this fluid flow acted as their mechanical stimulus (shear stress). By calculation of the signal generated by every osteocyte in response to the fluid-induced shear stress, this model predicted structural changes for a single trabecula subjected to cyclic loading with various frequencies.

The recent efforts of Adachi and colleagues were focused on incorporation of mechano-dependent intercellular signalling into the described model [73]. Osteoblasto-/osteoclastogenesis and osteoblast apoptosis were regulated by concentrations of signalling molecules produced in response to the stress-nonuniformity stimulus. The modified algorithm was used to analyse morphological changes in a 3D FE model of trabecular lattice obtained from murine micro-CT scans. Subjected to physiological loading, this model demonstrated adaptive reorganization of trabecular architecture for the load support. Conditions of the load-induced osteoporosis and osteopetrosis were modelled in terms of the decrease and increase in the applied loading, respectively. The osteoporotic model demonstrated an initial decrease in BV/TV, which plateaued after two weeks of simulated adaptation. The opposite process was observed in the osteopetrotic case.

### 2.4 Model D – Daily stress

The model of Carter et al. [9,50] predicts BA using the daily stress stimulus (Eq. (15)) that accounts for the effective stress (Eq. (14) and the number of daily cycles. This model determines the remodelling rate according to the function presented in Fig. 1(v). When the value of the daily stress stimulus exceeds the lazy-zone conditions, bone-resorption and -formation activities are initiated, with the remodelling rate changing along the corresponding slopes (Eq. (16)).

Model D was extended to introduce overload-induced bone resorption for prediction of BA around dental implants [74, 75]. The latter study demonstrated bone-loss values within the ranges observed in longitudinal *in vivo* human scans for the corresponding lengths of the implants. In addition, this model was employed in FE models of human femora, which had the same geometry and boundary conditions but differed in the values of initial density [76]. Different equilibrium distributions of density were obtained with these models, indicating the problem of non-uniqueness of the solution. This difference was eliminated by introduction of a “lazy point” instead of the lazy zone into the remodelling curve, with the stimulus values in the range of *F_ref_* ± *w* (Fig. 1(v)) corresponding to a low but nonzero rate of remodelling [77].

Model D was further improved by introduction of dependence of the daily stress stimulus on loading frequency [78]. The number-of-cycles variable in the original Eq. (15) was replaced with the Darcy velocity that considers the lacunar-canalicular fluid flow. When used in conjunction with a 3D FE model of animal ulna subjected to several loading cases, this algorithm reported loading frequencies of 5-10 Hz that caused the highest increase in bone remodelling rates (around 200 µm/year and 220 µm/year, respectively), corresponding well with the experimentally observed remodelling rates (200 µm/year and 170 µm/year, respectively) for the same loading amplitude (2 N) [79].

## 3 Comparison of bone adaptation models

The BA models reviewed in this report were demonstrated to predict structural and density changes in TB caused by various loading conditions. Although the exact nature of the mechanical stimulus that drives BA is not clear, these models considered previous experimental and simulation findings to explain the choice of the stimuli used. The stimuli employed in the Models A, B and D are the products of both stress and strain tensors and are likely to efficiently represent the response of bone material to loading. In Model C, the authors did not explicitly mention what stress component they use, making its comparison with other mechanical quantities difficult. Another factor to consider is that, in daily activities, long bones are mostly loaded in bending [80], giving rise to the stress and strain gradients in them. These gradients are better accounted for by the stimuli that consider the stress/strain nonuniform distributions on the bone surface, e.g., the stress-nonuniformity stimulus in Model C. For example, the SED-gradient stimulus better predicted the formation and resorption activities as compared to the SED stimulus [40]. In contrast, TB's microstructural parameters derived from the maximum-principal-strain stimulus better correlated with experimental data than those derived from the maximum-principal-strain-gradient stimulus [81].

Introduction of parameters of loading history such as number of cycles, loading frequency and microdamage accumulation into the BA models allows the simulation of such scenarios as under-/overload and cyclic loading. All the reviewed models incorporate a similar effect of inactive bone by initiation of resorption when mechanical stimuli are below the respective threshold value. The overload is represented differently in the reviewed models. Model A accounts for the overload-induced resorption but does not consider the number of loading cycles. Model C considers neither the number of loading cycles nor microdamage accumulation. Model D accounts for the number of loading cycles but neglects the formation of microcracks. The suitability of these models for the cases of a complicated real-life loading is, therefore, questionable. Model B considers both the number of cycles and microdamage accumulation, which makes it suitable for prediction of BA in overload. Cyclic loading is better represented by Model D that accounts for the number of cycles and the loading frequency, where repeated loading condition is the subject of the study.

The theory of mechanical-stimulus transmission from the in-depth osteocytes to the bone surface is believed to occur via the release of several signalling molecules [17] and calcium signalling propagation from the mechanosensory cells to the bone surface cells [82]. Models A, B and C incorporate the osteocyte-influence-distance variable that modulates the decay of a stimulus transmitted to the bone surface cells. The levels of osteocyte influence distance used in these models are comparable to the experimentally found calcium signalling distance of few hundred micrometres [83]. In contrast, Model D does not consider the transmission of a mechanical stimulus to the bone surface, which may decrease its accuracy in predicting resorption and formation sites in complex trabecular microstructures.

All the described adaptation models include time dependency in the calculations. Yet, definition of time in these models is not uniform and sometimes could be contradictory. In many studies it is not directly related to specific time intervals of days or weeks. Most studies that used Models A [84], B [85], C [49] and D [74,75,86,87] related one iteration of the simulation to one day of a real-life BA process. However, one study that used Model A for simulation of the osteoclasts’ resorption activity set one iteration equal to 1/4 day [52], while another study that implemented Model C to predict the adaptation of TB lattice reconstructed from micro-CT scans set the iteration time equal to 12 days [65]. The difference in setting the time-dependent remodelling activity rates means that a cross-model comparison of the key simulation parameters from different models using various mechanical stimuli, loading conditions and the anatomical locations is difficult. Should such a comparison be made, the time-adjusted remodelling rate per iteration must be considered accordingly.

Although the lazy zone hypothesis, where a physiological range of the stimulus magnitudes produces no significant adaptation response, was incorporated into all described models, it is not supported by all researchers. For example, a continuous linear relationship between the applied load and the sites of BR was found in animal tibiae and vertebrae [88,89] as well as human tibiae [27]. There is a tendency of using Model D without the lazy zone in recent studies, which improves stability of the equilibrium BMD distribution in simulations.

A summary of the factors considered in the reviewed BA models is given in Table 2.

**Table 2:** Comparison of reviewed BA models.

Model	***A***	***B***	***C***	***D***
Model name	Strain energy density	Strain and damage	Stress nonuniformity	Daily stress
Mechanical stimulus	SED	SED	Stress	Effective stress
Lazy zone	+	+	+	+
Overload-induced resorption	+	+	–	+
Damage accumulation	–	+	–	–
Transmission of mechanical stimulus to bone surface	+	+	+	–
Osteocyte influence distance, μm (decay function)	≈200-400 (Exponential)	≈200 (Exponential)	250-1000 (Linear)	N/A
Reduction of osteocyte influence distance in overloading	+	–	–	N/A
Nonuniformity of mechanical stimuli on bone surface	–	–	+	–
Number of cycles	–	Only for damage stimulus	–	+
Loading frequency	–	–	+	+
Iteration time, days	0.25-1	1	1-12	1

## 4 Conclusion

This report discusses the recent progress in *in silico* modelling of mechanoregulated TB adaptation. Reviewed BA models incorporate different aspects of the real-life BA process, which make each of them more suitable for simulation of specific loading scenarios. Some factors influencing mechanoregulated BR are widely accepted by researchers and incorporated into the reviewed adaptation models: loading configurations (number of cycles, loading frequency and, additionally, the amplitude of the dynamic loading cycle [79]), microdamage-induced resorption, transmission of mechanical stimuli to the bone surface and distance-dependent decay of a stimulus. However, some aspects of the load-induced BR are still controversial, such as the exact nature of the BA stimulus and the presence of the lazy zone in the remodelling curve.

There are several major disadvantages of the described *in silico* approaches that limit validation of their results against *in vivo* longitudinal CT studies. First, some of the described studies modelled TB using 2D geometry only, whereas TB is a complex 3D structure which cannot be accurately represented in 2D. Second, many works presented TB as isotropic homogeneous material, with the trabecular microstructure neglected. Third, artificially generated trabecular architecture used in many studies may not always represent the real-life TB microstructure. One of the solutions is to use the reviewed adaptation algorithms in conjunction with FE models of TB reconstructed from high-resolution CT scans. This can allow tracking of local changes in BMD and trabecular microarchitecture and their comparison with clinical radiological studies, which is especially important for prediction of the individual-trabecula remodelling. Furthermore, neural networks can be used for generation of representative volume elements that retain morphological characteristics of TB. In addition, introduction of more complex loading regimes into FE simulations, corresponding to the real-life bone loading conditions, can also contribute to prediction of more realistic TB morphologies.

Despite the described limitations, *in silico* approaches are a useful tool for investigation and characterisation of the mechanisms underlying mechanoregulated BR. Over the last decade, BA models became more mathematically sophisticated, with their predictive accuracy improved thanks to incorporation of the recent discoveries in bone biology. From recent developments it is clear that some research directions converge, such as related to the understanding of damage/microcracks and the distance-dependent decay of mechanical stimuli. On the other hand, some areas diverge, such as description of the lazy zone and definition of mechanical stimuli. Still, these latest improvements will contribute towards further development of *in silico* models for better estimation of patient-specific BA in different loading conditions and evaluation of the risks for patients affected by bone pathologies.

## Declaration of Competing Interest

The authors declare that they have no known competing financial interests or personal relationships that could have appeared to influence the work reported in this paper.
